# Utilizing population variation, vaccination, and systems biology to study human immunology

**DOI:** 10.1016/j.it.2015.06.005

**Published:** 2015-07-14

**Authors:** John S. Tsang

**Affiliations:** 1 Systems Genomics and Bioinformatics Unit, Laboratory of Systems Biology, National Institute of Allergy and Infectious Diseases (NIAID), Bethesda, MD 20892, USA; 2 Trans-NIH Center for Human Immunology, Autoimmunity, and Inflammation (CHI), National Institutes of Health, Bethesda, MD 20892, USA

## Abstract

The move toward precision medicine has highlighted the importance of understanding biological variability within and across individuals in the human population. In particular, given the prevalent involvement of the immune system in diverse pathologies, an important question is how much and what information about the state of the immune system is required to enable accurate prediction of future health and response to medical interventions. Towards addressing this question, recent studies using vaccination as a model perturbation and systems-biology approaches are beginning to provide a glimpse of how natural population variation together with multiplexed, high-throughput measurement and computational analysis can be used to uncover predictors of immune response quality in humans. Here I discuss recent developments in this emerging field, with emphasis on baseline correlates of vaccination responses, sources of immune-state variability, as well as relevant features of study design, data generation, and computational analysis.

## Introduction

The immune system is extraordinarily diverse, with a wide range of cell types in distinct states of differentiation and activation residing in almost all tissues and organs. This diversity in the composition, location, and molecular state of immune cells also vary both within and across humans (intra- vs inter-individual variations, respectively). Over the past few decades immunology has primarily focused its efforts on reductionist approaches that examine individual molecular and cellular immune components using powerful *in vitro* and animal models. By contrast, the status of the immune system as a whole, both within and across human subjects, is less well studied [1,2] in part owing to the staggering number of potentially relevant immune parameters, including gene expression programs within individual cells, the frequency and location of cell subsets, as well as the level of circulating molecules including cytokines, chemokines, and growth factors. Fortunately, recent advances in data acquisition, including high-throughput, multiplexed technologies such as transcriptome and proteomic profiling [3,4], DNA sequencing [5], and single cell technologies [6–8], together with new computational approaches for analyzing, integrating, visualizing and modeling such datasets [9–17], are starting to provide an increasingly detailed view of human immune states and responses at multiple scales. In particular, deep assessments and computational analyses of immune states in blood samples collected before and after vaccination have been productive first applications of such ‘systems’ approaches to understanding human immunity; such studies are beginning to yield novel correlates of vaccination outcome, insights into mechanisms of vaccine action, and initial assessments of inter- and intra-subject variations both before (baseline) and after vaccination [13,18–23].

**Figure 1:**
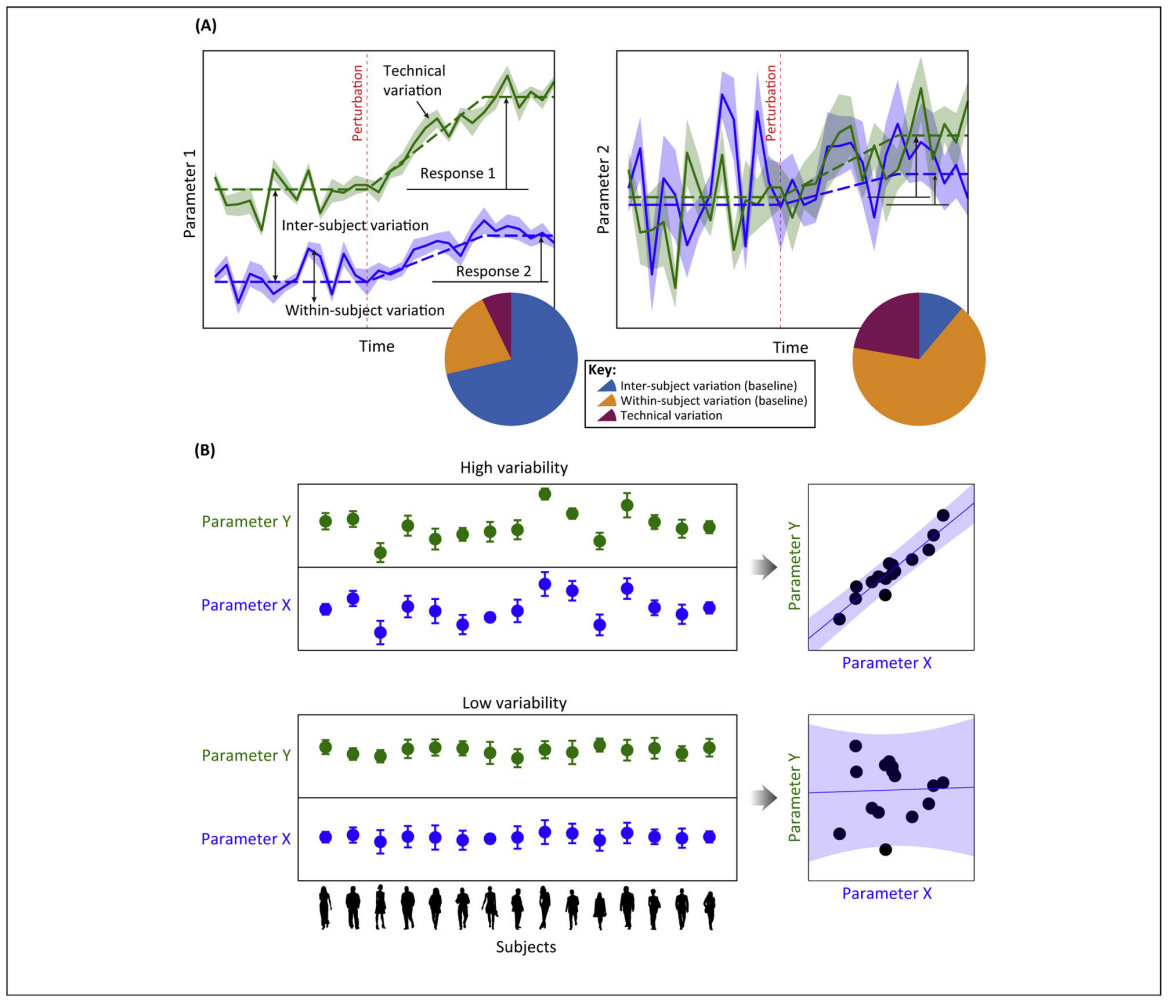
**(A)** Illustration of the dynamical trajectory of two hypothetical parameters within two subjects (green and blue lines) before and after a perturbation. At any given moment (e.g., a snapshot measurement of the parameter in both subjects) before the perturbation (i.e., ‘baseline’), the total amount of observed variability can be attributed to inter-subject differences, temporal variations within subjects, and technical variation (or measurement noise – as indicated by the thickness of the shade). The measured variation in parameter 1 (left panel) is dominated by inter-subject variation (decomposition of variation is illustrated using a pie chart), and thus is an example of a temporally-stable parameter – in other words, inter-subject difference is well maintained regardless of when the measurement is made. Parameter 2 (right panel) exhibits lower subject-to-subject differences, but the fluctuations within subjects are much higher relative to parameter 1. Parameter 2 is an example of a temporally-unstable parameter. Both subjects responded to the perturbation by increasing the value of parameter 1, but the amount of increase relative to the baseline is different across the two subjects, thus showing a qualitatively-consistent change (i.e., a coherent change) that is quantitatively variable (i.e., a response variation). Parameter 2 also showed an increase in value after the perturbation but, owing to the amount of fluctuations within subjects, this coherent change is difficult to detect statistically. **(B)** Extensive subject-to-subject variability is essential for assessing correlation between two parameters. The top panel illustrates a scenario where two biologically associated parameters X and Y exhibit substantial subject-to-subject variability relative to measurement noise (indicated by error bars), which in turn enables robust detection of correlation between these two parameters (right scatter plot). The bottom illustrates an opposite scenario: the two parameters here are also biologically associated (i.e., they co-vary) but, because the amount of inter-subject variation is small relative to measurement noise, detection of correlation is impossible (right scatter plot).

In addition to genetics, the immune system is subject to the environmental influences including those from diet, commensal microbes, infections, and pathological perturbations such as cancer [24–28]. Such environmental perturbations can shape the generation and expression of clonally distributed, variable receptors in lymphocytes, as well as molecular and cellular phenotypes including epigenetic states, gene expression programs, and trafficking in populations of immune cells. Thus, the genetic diversity of the human population, together with the varied environmental exposures and life-histories of individuals, give rise to highly diverse immune states (Figure 1A). Beyond ethical issues and cost factors, the existence of this diversity has played a major role in tempering the enthusiasm for conducting experimental studies of the immune system in humans – with good reason; for example, responses to therapeutic interventions can be highly variable and therefore less conclusive compared to studies using inbred animal models under stringently controlled conditions [2].

However, genetic and phenotypic variations can be advantageous in studying humans where experimental interventions are often limited because these natural ‘perturbations’ are essential to robustly establish correlations among parameters [13,29] (Figure 1B). Such correlation analysis can uncover predictors of clinically-useful endpoints, underpin the construction of models of functional interactions among immune parameters, and, ultimately, enable inference of quantitative intra- and intercellular networks that can help generate mechanistic hypotheses for further testing. Indeed, recent population-based, systems-level studies of vaccination are beginning to reveal the power of utilizing variable human immune states to uncover both mechanistic insights and potential biomarkers for predicting immune-response quality [10,13,18–21,23,30–32]. In turn, vaccination provides a systemic perturbation for studying human immunity that is applicable in diverse settings and populations [33].

Assessing and understanding human immune-state variability before and after a perturbation is pertinent beyond vaccination, particularly in light of the recently launched, large-scale effort to move toward personalized, precision medicine [34]. Given the involvement of the immune system in diverse pathologic states [1], an important question is how much and what information about the state of the immune system is required to enable accurate personalized prediction of future health and response to medical interventions. Several recent systems studies, which will be covered in more depth below, suggest that a substantial fraction of immune-state variability, including but not limited to that of vaccination responses, cannot be solely attributed to genetics. Thus, comprehensive immune monitoring may be needed to complement other large-scale, ‘omics’ data to achieve the goals of a more personalized and precise medicine.

This review discusses emerging findings involving human vaccination studies employing systems biology approaches. Emphasis will be placed on (i) providing a synthesis of emerging pre-vaccination or baseline correlates uncovered in recent studies that can explain vaccination response variability across human subjects; (ii) discussing the sources of immune-state variability – with particular emphasis on genetics versus other contributors – and how these have been utilized to assess relationships among immune variables; and (iii) highlighting relevant features of study design, data generation, as well as computational analysis and modeling (Boxes 1 and 2). Post-vaccination response variability and correlates involving molecular and cellular parameters will also be discussed, albeit briefly by touching on the latest developments because major findings in this area have already been covered in other reviews (e.g., [30,33,35]).

Box 1 **Study design and technology considerations**

**Cohort characteristics**

Thus far the cohorts used in vaccination studies using systems approaches are either composed of mostly younger adults (<40 years of age) (e.g., [13,19]) or a mixture of younger and older subjects to study age-related effects (e.g., [18,82]). Studies involving the very young and extremely old are more rare (e.g., [107]), but upcoming studies should help fill this gap (personal communications). Given the contribution of various intrinsic variables as discussed here, an important study-design consideration is to balance the subject characteristics based on the primary variables of interest. For example, if the goal is to assess the baseline and vaccination responses in the old versus young, the gender and race of the study subjects should be balanced between the two age groups (e.g., the number of subjects of each gender should be similar between the old and young groups). Another consideration is whether to utilize discrete, ‘extreme’ ends of the variability spectrum. For the age example, one potential design is to compare two groups of subjects with largely non-overlapping age distributions (e.g., young adults vs very old), or to recruit a cohort with a more natural, continuous distribution of ages. An extension of the latter strategy is a fully ‘balanced’ design in which the distribution of different variables resembles that of a targeted distribution, for example one that captures the distribution of the study population [108]. The potential advantage of the ‘extreme’ design is that, because the differences between the ends of the distribution may be large, it is more amendable to using smaller sample sizes. However, the balanced design might be more appropriate for hypothesis generation to uncover different types of relationships among variables, but larger sample sizes are needed.

**Sampling strategy: time-points**

If possible, samples obtained from at least two pre-vaccination or baseline time-points spanning a reasonably long period should be collected to help to assess the temporal stability of measured parameters; more time-points may be needed to increase robustness (e.g., [13,23]). In terms of the response, most vaccines seem to perturb a relatively small number of immune parameters, in other words those that change in a consistent manner across subjects (e.g., a few to tens of cell subsets, some of which are related; hundreds of transcripts in PBMCs or whole blood [10,13,19,20,22]), but are likely dependent on measurement timing (see main text). Because patterns of individualistic responses have been detected [40], new patterns of responses could emerge as the temporal resolution of sampling is increased in a larger number of subjects. In general, samples from days 1 or 3 post-vaccination are used to assess innate responses – indeed, changes involving innate cells and general inflammatory transcript signatures have been detected at these early time-points, although strong signals from adaptive cells and processes have also been detected, perhaps owing to recall, memory responses [13,35]. A day 7 sample is usually used to assess the adaptive response, largely because the peak of the plasmablast response tends to occur around that timeframe [35,109]. However, as discussed in the main text, this can also occur earlier or later. In general, response timing and duration variation across subjects can be a major source of heterogeneity, and therefore dense temporal measurements will be an important element of future study designs.

**Sample types and assays**

Broadly speaking, two types of analyses are carried out using cells extracted from blood. One is to assay the ‘natural’ state of cells and the other is to perform *in vitro *perturbation to the cells and assess their responses, for example, by using transcript profiling or flow/mass cytometry to assay responses [7]. Both types of analysis can be carried out using whole blood (e.g., using syringe-based assay systems [110]), PBMCs, or, in some cases, isolated or sorted cell subsets such as CD8^+^ T cells [86].

Gene expression profiling has been performed typically using whole blood Paxgene or Tempus tubes (e.g., [23]; also for PBMC stimulation assays) or total RNAs extracted from PBMCs or sorted cell subsets (e.g., [19]). In both cases, RNAs can be preserved with high quality for relatively long periods using appropriate reagents. In addition to its ease of use, the potential advantage of profiling from whole blood is that, in principle, the transcriptomic state of freshly isolated cells is preserved without the need for additional processing as in the preparation of PBMCs. However, depending on the goal of the study, in principle signals from rare cell subsets may be more difficult to detect in whole blood compared to PBMCs owing to the large fractions of neutrophils present in whole blood, most of which are lost during PBMC preparation. Similarly, profiling of sorted cell subsets can further enrich signals from less-frequent cell subsets, but could suffer from undesirable signals originated from cellular stress associated from cell isolation or sorting.

Microarrays and RNA-seq are both capable technologies for obtaining transcriptomic profiles containing information on the relative level of transcripts genome-wide (note that neither provides absolute copy measures) [111–113]. While the latter is still more expensive, cost is coming down, and it potentially offers better sensitivity when appropriate sequencing depth is achieved, as well as additional information on, for example, splicing isoforms, RNA editing, and alternative promoter usage. In general, a broad transcriptomic survey for enabling differential expression analysis of genes with medium to high expression levels can be robustly achieved with ~30–40 million reads per sample. Longer (100–150 bp), paired-end reads and greater sequencing depth can provide more robust detection of splicing isoforms. Currently, however, analysis of RNA-seq data can entail a higher bioinformatics overhead and involves using less-mature methodologies compared to microarrays.

Recent advances in flow- and mass-cytometry have enabled the simultaneous analysis of tens of protein parameters at the single cell level. These techniques can be used to assess the frequencies of diverse immune cell subsets across distinct cell lineages in fresh blood and in PBMCs. More importantly, standardization efforts in flow cytometry have resulted in the creation of lyophilized plates adopted by multiple institutions to reduce technical variability and increase data comparability across studies and laboratories [84]. Regarding mass-versus flow-cytometry, each technology has its pros and cons. The readers can consult recent reviews on this issue (e.g., [7,114]).

Single cell expression-profiling technologies, such as single cell RNA-seq and quantitative PCR, can measure the entire transcriptome or ~100 transcripts within individual cells, respectively. They are still relatively expensive and not yet mature enough for the profiling of tens of thousands of immune cells per sample as is routinely performed using flow cytometry. However, these technologies are progressing rapidly; for example, most recently droplet-based approaches have been shown to be capable of assaying tens of thousands of cells with relatively low cost [90,91]. Thus, further refinement of such technologies should enable efficient and low-cost profiling of diverse immune cells within and across human subjects, although one major bottleneck that will likely take more time to overcome is assay sensitivity. The number of transcript copies per cell for a typical gene is generally quite low: ~17 based on one recent estimate [115], and can go down to a few copies for genes such as those encoding transcription factors. Thus, perfect detection down to individual molecules for all assays in a multiplexed setting is difficult to achieve, partly because it has been challenging to substantially improve the enzymatic chemistry of the reverse transcription reaction for capturing minute amounts of RNA [116].

Preservation of fresh blood for future experiments is, in general, more challenging compared to that of PBMCs. Cryopreservation of PBMCs is central to many human studies because it often enables appropriate batching schemes to help mitigate batch effects (see below). Because results obtained from fresh versus frozen PBMCs are not always comparable [117], adopting existing assay standards together with a thorough evaluation of technical variations before a study begins would be ideal to assess the pros and cons of different approaches.

In addition to cells, serum or plasma are typically assayed using proteomic technologies such as bead-based technologies (e.g., Luminex) [118,119], classical ELISA, or, more recently, DNA-based aptamers [120] to measure the level of cytokines, chemokines, growth factors, peptides and proteins. As in any multiplexed assay, the technical performance is target-dependent and can vary substantially from vendor to vendor in terms of plate-to-plate, well-to-well, and reagent lot-to-lot variability. For example, the intensity distribution of coated beads in Luminex assays can have unexpected multi-modal distributions that render commonly reported statistics such as the mean or median fluorescent intensity (MFI) across beads difficult to interpret [121]. Thus, improved low-level data-processing approaches that explicitly model such variability need to be developed [121].

**Assay batching**

Batch effects are ubiquitous in most if not all high-throughput, multiplexed assays used in systems studies. Thus, major batch variables of each assay need to be determined (e.g., operator, reagent lot, machine). Depending on the study design, samples should be randomized and batched appropriately. For example, if comparison of samples from the same subject is essential, cryopreservation of sera and PBMCs followed by the processing of all samples from the same subject in one batch is essential (e.g., [13]). For example, if age and gender effects are large and are key variables in a study, randomizing study subjects to avoid having all older individuals processed in one batch is essential to avoid confounding between age and batch effects. Ideally, important meta-data about assays and experiments (e.g., operator, reagent lot) should be recorded systematically such that batch effects can be accounted for in downstream data processing and modeling.

**Sample size**

A common question is what sample size is needed in a study. This is a challenging question to answer because many variables are assessed together in systems-immunology studies, each of which has different levels of technical variability; and the biological effect, and the effect size, of the perturbation on most of these variables are often not known and are in fact waiting to be discovered through the study itself – in other words it is a ‘chicken and egg’ problem in that it would be difficult to determine what sample sizes are needed without *a priori *knowledge of the magnitude of changes induced by a perturbation. In addition, the key parameters used in classical statistical power calculations, including the type of statistical test used, effect size, and technical noise [108] are even less well understood for assessing correlations among variables, which is a major goal as discussed throughout this review. The tens to 100+ subjects used in existing studies are certainly on the lower end of what is required given the substantial technical variability in most of the assays [35]. While existing studies can clearly detect major changes after vaccination, uncovering predictive parameters from the tens and thousands of whole blood or PBMC transcript measurements has been challenging, aside from the identification of age- or plasmablast-related predictive signatures, which clearly have sizable effects on vaccination outcomes. However, for example, pre-vaccination/baseline predictive signals related to adjuvant-like, ‘innate’ type of signatures have not been robustly detected, likely due to statistical power issues. Fortunately, larger studies are on the horizon [35].

Perhaps one potential design scheme is to plan studies adaptively up front: first carry out a sufficiently large study to obtain initial assessment of effect sizes, technical variability, and the identity of parameters that may be linked to the end-point being studied – even those with weak signals that do not survive multiple hypothesis testing correction can be considered further, especially if they are biologically compelling based on other information. This initial phase can be followed by assaying additional samples/subjects and perhaps also by using lower-throughput, but technically less-noisy assays to measure a subset of key variables. Systematic, iterative application of this scheme may help to overcome the initial lack of quantitative information for estimating sample size requirements, and at the same time can enable systematic discovery and validation of novel correlates and predictors in a relatively unbiased manner. Future improvements in assay sensitivity and technical noise may reduce sample size requirements. Finally, meta-analysis across multiple studies can lead to better estimates of biological effect sizes and thus enable better power calculations, as well as better biological discoveries, by robustly pooling signal across multiple smaller studies.

Box 2 **Data analysis and integration considerations**

**Antibody titer response definitions**

In most studies the level of post-vaccination antibody titer as indicated by HI or neutralization assays is used as a primary response end-point. For vaccines such as YF-17D most subjects are naïve before vaccination, therefore the fold-increase metric would work well. However, as discussed in the main text, because pre-existing immunity is prevalent for influenza, the fold-change metric can exhibit a non-linear correlation relationship with the pre-existing antibody titers before vaccination (see Figure 4A in [13] for an example of such a non-linear, ‘triangular’ relationship). Thus, pre-vaccination titer alone is often a strong correlate of the response defined by the fold-change in titers. Further complicating the matter is that the seasonal vaccine contains antigens from three influenza strains. Thus, the maximum fold-change across the three virus strains was used in both Nakaya *et al. *and in our study [13,19] because none of the molecular and cellular parameters we assessed reflected specificity to any of the strains.

To define a metric that is statistically independent of pre-existing titers, in other words to capture the response variability among individuals who had similar pre-vaccination titers, two general approaches have been devised. The first, used in Bucasas *et al. *[21], employs linear regression to model the relationship between the pre-vaccination titer and its fold-change after vaccination; the residual – in other words, the variation remaining after the regression fit, or the difference between the fitted value and the actual datapoint – was used as the new vaccination response end-point. However, linear regression cannot fully remove the non-linear relationship.

In our study we opted to focus on defining response metrics that capture the relative response across individuals instead of treating the titer values as absolute measurements (e.g., this approach can better mitigate the effect of noise in titer measurements, which can be substantial [35]). We therefore computed the relative response across the cohort for each virus strain before taking the maximum followed by removing the non-linear relationship between initial titer and its fold-change by a binning procedure (for details see Supplemental Information in [13]). The resulting metric, called ‘adjusted MFC’ (adjMFC), captures the relative response across individuals who had similar initial maximum titers across virus strains (i.e., capturing high vs low responders as opposed to ‘responders’ vs ‘non-responders’). We are in the process of releasing an R package for computing this and related metrics from titer data (Y. Kotliarov and J.S.T., in preparation).

**Source of variation: inter versus intra versus technical variations (and temporal stability)**

To assess the relative proportion of inter-subject versus temporal (within-subject) variations, measurements from multiple time-points without overt perturbation are needed (e.g., from multiple pre-vaccination time-points). The total observed variation in a measured parameter in such a dataset can be attributed to at least three sources (Figure 1A): (i) subject-to-subject variation, (ii) variation within a person over time, and (iii) technical or measurement noise. To estimate contribution from the third, each sample has to be measured multiple times (i.e., technical replicates), but such data are often not available. Thus, while one can directly estimate the fraction of observed variation attributable to inter-subject differences (because subject labels are always available), the remaining unexplained variation could be a combination of both within-subject/temporal and technical variations. One approach to address this issue is to estimate the technical variability separately by assuming that technical variation stays relatively constant across experimental batches. For example, in our study [13] using separate technical experiments we found that the flow-cytometry assay used to quantity cell subset frequencies exhibited very small technical variability, and thus most of the remaining variation after accounting for subject-to-subject differences was unlikely to be due to technical variations, and therefore more likely reflects temporal variations within subjects. ANOVA or mixed-effects modeling can be used to perform such variability analysis [13,122].

**‘Untangling’ of correlates and predictors**

**Figure 2:**
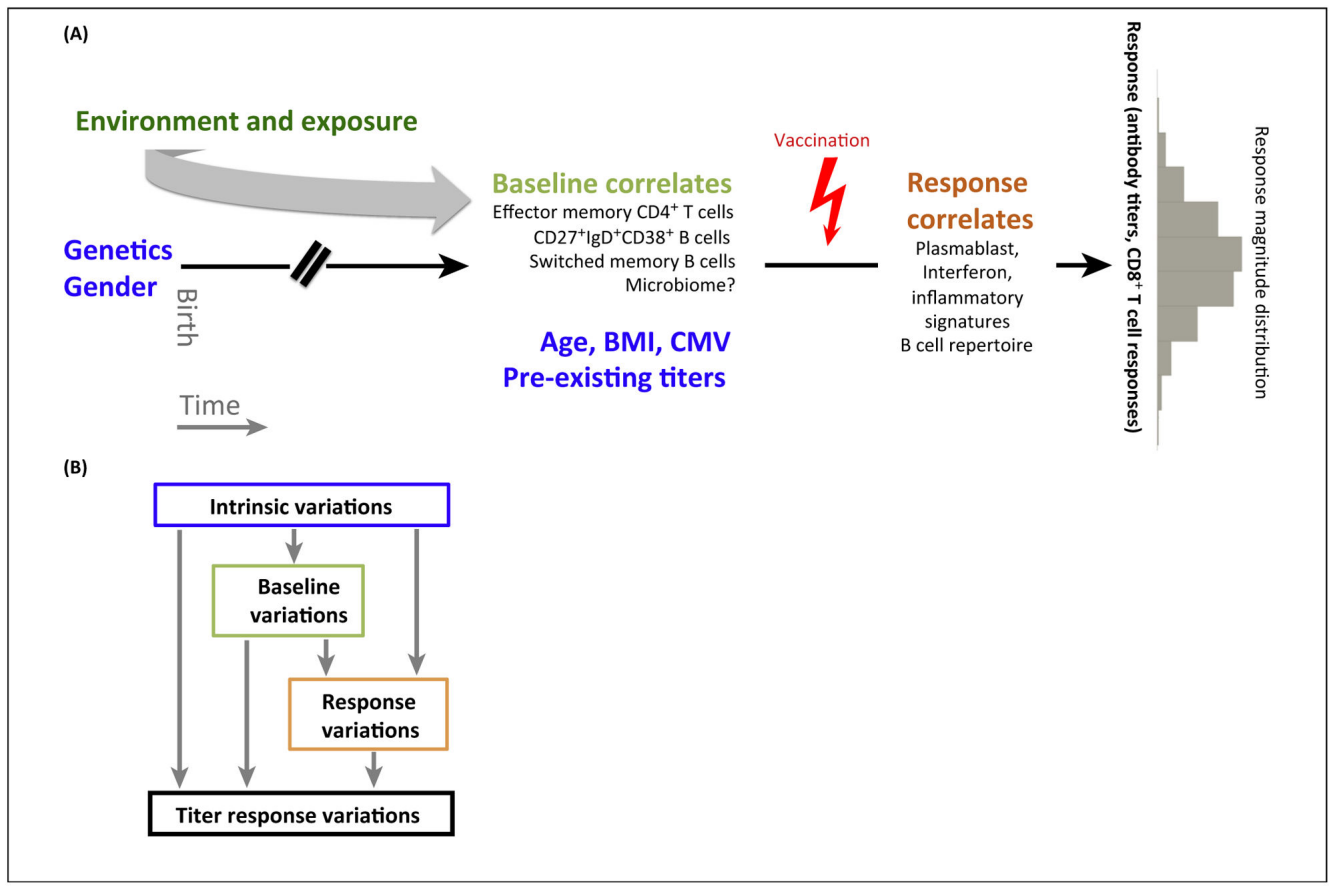
Contributors to vaccination response variability. **(A)** Genetics, gender, and accumulated environment exposure since birth shape the pre-vaccination state of the immune system which, together with other intrinsic variables such as BMI, age, status of chronic infection (CMV/EBV), and initial antibody titers, can help to explain inter-subject variability in antibody titer responses, whose distribution across subjects is depicted as a gray histogram on the right. In addition to the known intrinsic correlates (highlighted in blue font), some of the pre- and post-vaccination correlates identified in recent studies are also indicated. **(B)** One approach for assessing contributors of titer response variation following vaccination. Intrinsic factors (e.g., age, gender) can contribute to variations in baseline immune states and post-vaccination responses. Baseline immune states can in turn contribute to response variations (Figure 1A), and all of these factors can together contribute to variation in titer responses. Correlates of titer responses can be analyzed following the ‘flow’ of time: the contributions of intrinsic variables are first analyzed, and their effects are then removed statistically from baseline and response parameters, which were then analyzed in a similar stepwise manner to assess their independent contributions to titer response variation. Figure design adopted from Figure 5A in [13]. Abbreviations: BMI, body mass index; CMV, cytomegalovirus; EBV, Epstein–Barr virus.

One issue in the context of developing predictive models and finding correlates is that both pre- and post-vaccination parameters can be correlated (or ‘entangled’) with each other. Thus, for example, given that intrinsic variables such as age and gender can be correlated to a sizable number of other parameters (e.g., frequency of Tregs at baseline), it is important to account for their effects on other variables to assess whether any potential correlate is a true ‘root’ correlate or whether the observed correlation was merely driven by cross-correlation with variables such as age and gender. When sample size is sufficiently large, one potential approach is to build a probabilistic graphical model to account for dependencies among all variables, from intrinsic to both pre- and post-vaccination parameters [123]. However, when sample size is relatively small, as in existing vaccination studies, another approach is to account for contributors to end-point variation in a stepwise manner, first by examining the correlation between intrinsic variables and the endpoint, then by analyzing other parameters over the progression of time – from pre- to post-vaccination time-points after removing the effect of intrinsic factors (see Supplemental Information in [13] for details). In such an approach, the variables analyzed first could affect subsequent variables, but not the other way around (e.g., baseline parameters cannot affect age, but age can affect baseline parameters) (Figure 2B).

**Dimensionality reduction and gene set based analysis**

Transcriptome profiling generates data on tens of thousands of variables, while other multiplexed assays such as flow cytometry tend to focus on tens or hundreds of variables. This gap in dimensionality is one barrier to effective integration of different data modalities. Reducing the dimensionality of gene expression data in a biologically meaningful way can better enable such integration, and can also lead to better biological insights. One popular approach for achieving this is module analysis [9,10,23,124]. Modules are sets of genes derived from analysis of co-expression patterns. In the Chaussabel approach [9], genes are first connected by co-expression relationship within individual datasets obtained from public gene expression databases, for example, expression data obtained from whole blood; highly connected clusters of genes supported by different degree of connectivity (in terms of the number of datasets/studies supporting the connection) are then identified as ‘modules’. In the Li approach [10], modules are formed by analyzing co-expression relationships across a large number of samples spanning multiple studies measured using the same gene expression platform. Co-expression relationships are inferred using mutual information, thus potentially capturing non-linear co-expression relationships. In both approaches, the derived modules can correspond to intra- and intercellular interaction networks, but also genes with enriched expression in particular cell types in blood (see below on deconvolution).

Once modules are derived from independent datasets, the module ‘activity’ or ‘score’ can be computed at the individual sample level using different approaches [13,125–127] (or simply taking the mean or median among genes as was done in [23], provided that the correlation among the genes in the modules is high) to generate a module-by-sample matrix. This matrix can then be used for downstream modeling and correlation analysis.

Owing to smaller magnitudes of change, assay noise, and insufficient sample sizes, some transcripts that are altered following perturbation may not be detected as statistically significant. Instead of focusing on individual genes, module- or gene set-based analysis can be used to potentially improve sensitivity by assessing coherent, qualitatively-consistent trends of alteration among genes from the same module using approaches such as gene set enrichment analysis [128,129].

**Correlation and prediction**

Correlation among variables can sometimes be driven by outlier samples, especially when the Pearson correlation coefficient is used. To ensure that correlations are robust against outliers, non-parametric measures of correlation such as the Spearman metric can be used. Moreover, jackknifing can be used to assess the robustness of an observed correlation using multiple random subsets drawn from the samples [13].

Prediction can be considered as a more stringent way of assessing correlations. Predictions are often made using machine-learning models that combine information from multiple variables (X1, X2, …, Xk) to make a prediction about the value of an unknown variable Y. Cross-validation analysis by training a model using a random subset of the cohort, and then testing the model with the unused portion of the data, is used to assess whether prediction can be made within the cohort. Such an exercise is generally performed over a large number of random ‘trials’ to see if robust prediction can be consistently achieved. When a model is built during each iteration, there is often a ‘feature selection’ step that chooses a small subset of the input variables, which are then combined mathematically via a function (e.g., linear discriminant analysis, support vector machines; see [130] for details) to map the value of the selected features into a predicted value for Y (e.g., high vs low responders). The ultimate test of a predictive model is to assess it prospectively in independent cohorts.

Although the accuracy (or sensitivity) and precision (or specificity) are important measures of the performance of a predictive model, these two aspects need to be considered simultaneously rather than separately because changes that improve (or worsen) one will inevitably worsen (or improve) the other – for example, in a binary classification scheme (e.g., calling high vs low responders), perfect sensitivity can be achieved by calling everyone a ‘high’ responder, but then specificity suffers. Conversely, sensitivity can be reduced to improve specificity. A commonly used metric that captures the performance of a predictive model, and that evaluates both sensitivity and specificity, is the area under the receiver operating characteristic curve (or AUC). A model capable of prediction above random noise has an AUC significantly above 0.5.

**Gene expression deconvolution**

Gene expression changes in PBMCs or whole blood can be driven by (i) changes in cell subset frequencies (e.g., upregulation in monocyte frequencies can result in higher TLR expression because TLRs are expressed in monocytes), (ii) changes in gene expression level within individual cell subsets, or (iii) both. In general, computational deconvolution can involve two distinct tasks. One is to infer cell subset frequencies from expression data [11,75,131], the other is to infer gene expression changes or profiles specific to particular cell subsets by integrating PBMC or whole-blood gene expression data and cell subset frequency information [11].

## Variability in vaccination responses

While the ultimate goal of vaccination is to increase protection in the event of exposure to an infectious agent, response quality or quantity is often measured using correlates of protection (e.g., the level of antibody titer against the antigen) as opposed to protection itself [36]. While some studies have conducted infection challenge studies following vaccination, particularly when no or only weak correlates of protection are known, such as in the case of malaria [37], unless otherwise noted discussion in this review will center on assessments involving correlates of protection.

The quality and/or quantity of an immune response to vaccination (QR) usually involve measurement of humoral and, in some cases, cell-mediated vaccine-specific immunity. Responses are often expressed as the post- versus pre-vaccination fold-change in, for example, the serum antibody titer or the frequency of antigen-specific CD8^+^ T cells against the target antigen(s), respectively [36]. For example, according to the US FDA *Guidance for Industry* document [38], having a hemagglutinin inhibition (HI) antibody titer of 1:40 against influenza (seroprotection) or a fourfold increase in HI titer following influenza vaccination (sero-conversion) are considered protective. Using such definitions for classifying individuals into responders and non-responders can work well, especially when the studied subjects are naïve to the vaccine antigen(s) such as in the case involving the yellow fever vaccine (YF-17D) [20,22]. However, mathematical transformation of the fold-change metric [39] or use of alternative formulations may be needed to account for pre-existing immunity (which is highly prevalent in the case of influenza) such that correlates not associated with pre-existing immunity can be uncovered [13,21] (Box 2).

The timing of the peak response is vaccine- and subject-dependent, and thus is a potential source of variability in the response measured across individuals [40]. For example, antibody titers following influenza vaccination have been assessed at different time-points in various studies, such as days 7 [13], 21 [40], 28 [18,19], and 70 [13]. Data from a study in which daily measurements were performed indicate that an increase in titers is apparent only a few days after vaccination, with the timing of the peak response showing a dependence on prior vaccination status [40]. Interestingly, we found that titer responses on days 7 and 70 can correlate well [13], suggesting that although the timing of the peak response is subject-specific, the relative response across a cohort can be reasonably consistent across time-points, and therefore can serve as a robust metric in the context of correlate identification (Box 2).

## Correlates and predictors of vaccination response

Measurement timing aside, many biological factors can contribute to QR variation. While ‘correlates’ and ‘predictors’ are often used interchangeably to refer to biological variables that associate with QR in a statistically significant manner, here I make the qualitative distinction that a predictor needs to be able to delineate, with reasonable accuracy, the seroconversion status (e.g., responder vs non-responder [19]) or the relative response (e.g., high vs low responder [13]) of a given subject (Box 2). The term ‘predictor’ can also be used to denote a mathematical or machine-learning model that combines information from multiple parameters for generating sufficiently reliable predictions [13,18–20].

Given that individual correlates may not be biologically or statistically independent of one another (e.g., association between gender and expression level of genes from the Y chromosome), it can be advantageous, both conceptually and analytically, to consider the contributions of distinct categories of variables to QR variation along the time dimension (Figure 2; Box 2). Uncovering correlates in this manner [13] can increase the chance of finding ‘root’ correlates rather than surrogates that merely reflect other variables such as age, gender, or the presence of latent viral infections, for example, cytomegalovirus (CMV) [41]. This approach can be particularly informative when working with a large number of parameters over multiple time-points, as is often the case in systems studies of human immunity.

### Intrinsic correlates

‘Intrinsic’ variables, such as age (unless noted, in this review ‘age’ refers to chronological age; biological age may be environment-dependent), gender, and genetics, capture inherent or slower-changing characteristics of an individual; in other words, they are relatively immutable compared to most environmental, molecular, and cellular factors, particularly within the timescale of typical responses to vaccination or other acute immune perturbation (days to weeks). Age, gender, and genetics aside, factors such as body mass index (BMI) and chronic infection status exhibit substantial variation in the human population, and can also be considered as ‘intrinsic’. Together, intrinsic factors are a major source of biological variability: recent studies that measured a large number of immune parameters revealed that many are indeed correlated with intrinsic factors. Given that many intrinsic factors are also associated with vaccination responses, an important question is through what immune parameters and processes does an intrinsic factor exert its effects on QR. Recent studies are beginning to provide some hint on this issue.

#### Age

Age is a well-known QR predictor. Based on a meta-analysis of HI responses involving tens of studies, influenza vaccination efficacy in the elderly is estimated to be at best 50% of that seen in young adults [42]; this trend is clearly detectable and statistically significant in recent systems studies of influenza vaccination (e.g., [13,18]), despite the relatively small cohort sizes and the inclusion of just a small number of older subjects in one of the cohorts [13]. Reduced responses in the elderly are not limited to influenza vaccination, but have also been observed in other vaccines such as those against Tdap/Td (tetanus–diphtheria–pertussis or tetanus–diphtheria) and the varicella–zoster virus where the duration of protection may also be reduced [43]. This broad trend across vaccines suggests that immunosenescence, including defects in dendritic cell activation and a reduction in repertoire diversity in T/B cell receptors, could be a key driver of the attenuated response in the elderly [43]. At the other end of the age spectrum, infants and children younger than 2 years of age typically also have attenuated antibody and memory responses to many vaccines [44], although older children (2–18 years of age) tend to have responses similar to young adults. Thus, vaccination responses tend to decrease at both ends of the age spectrum.

A better understanding of the correlates and potential mechanisms of age-related defects in vaccination responses is needed to move toward better vaccine designs for the elderly [43,45]. Systems-level analyses are beginning to shed some light on this issue. In a broad analysis of baseline immune states in older (>60 years) and young (20–30 years) subjects [18], several pre-vaccination parameters with age-related variation were found to be predictive of the post-vaccination seroconversion status. Not surprisingly, the most informative predictor was age itself, and a majority of the other predictive parameters were significantly associated with age, suggesting mechanisms by which age could mediate its effect on vaccination outcome. These predictive parameters include: (i) the level of pre-existing antibodies and their reactivity profile against hemagglutinin peptides; (ii) the expression of certain genes and gene modules in whole blood, including modules associated with apoptosis (see Box 2 for module analysis); (iii) the frequency of particular cell subsets such as CD4^+^ effector and CD8^+^ central memory T cells; and (iv) signaling status of immune cell subsets, for example, phosphorylated STAT1 (signal transducer and activator of transcription 1) levels in activated CD8^+^ T cells. Interestingly, prediction using both baseline immune parameters and age did result in better performance compared to the use of age alone. However, when only baseline immune parameters were used the performance was similar to when only age was used. Together, these findings suggest that biological age is perhaps the ‘root’ correlate of vaccination response in such a cohort with bimodal age distributions (young vs older), whereby some of the predictive parameters capture aspects of biological aging not reflected by chronological age alone.

#### Pre-vaccination antibody titers

Pre-existing immunity against vaccine antigens is prevalent as a result of repeat infection and immunizations such as those involving influenza. The level of pre-vaccination antibody titer against influenza is highly variable in the population and has been found, both in vaccine trials [39] and in recent systems studies [13,18,21], as a strong negative correlate of antibody responses as assessed by the post-versus pre-vaccination fold-change in titers: subjects with higher initial titers tend to show lower proportional increases in titer than those with low initial titers, suggestive of a ‘ceiling’ effect. The mechanism behind this observation is not entirely clear, but rapid, broad neutralization of the vaccine components is a possibility. We also saw that individuals with higher levels of pre-existing immunity against influenza (indicated by higher initial antibody titers and influenza-specific B cell frequencies) tended to mount a lower plasmablast response by day 7 [13]. However, the extent to which the timing of the plasmablast response plays a role in this finding is unclear [40] (see below). Similarly to age, pre-vaccination titer was found to be correlated with a sizable number of pre- and post-vaccination parameters, including expression of genes in peripheral blood mononuclear cells (PBMCs) or whole blood, pathway status, and cell subset frequencies [13,18]; in turn, some of these were correlated with the titer response [13,21]. Thus, accounting for the effect of pre-vaccination titer is needed for uncovering correlates and predictors that are independent of pre-existing serology (Box 2). It will also be informative to further assess the extent to which some of the known influenza vaccine-response correlates (e.g., those from [10,19,35]) are linked to the level of pre-existing antibodies.

Despite its popularity, the fold-change in titers may not be the most informative metric for capturing some of the mechanisms through which a serological response is generated – for example, is the absolute level of increase a better reflection of the immunological response? After all, one needs to generate more antibodies for a twofold increase from an initial titer of 1:1024 than for a 10-fold elevation from 1:20. It would be interesting to see whether applying response metrics not based on the fold-change can lead to a distinct set of correlates and predictors.

#### Chronic and latent infections

In addition to pre-existing immunity against the vaccine-targeted pathogen(s), latent or chronic infection status, such as that involving CMV or Epstein–Barr virus (EBV), can affect vaccination responses. In a recent analysis Furman *et al.* saw an age-dependent effect of CMV infection status [41]: the presence of CMV infection tends to correlate with enhanced responses in the young but not in older subjects; this correlation was also observed in an inbred mouse model. In young but not older subjects CMV infection status was associated with an increase in the level of circulating cytokines indicative of T cell activation (e.g., IFN-γ and IL-13), higher frequencies of CD4^+^ and CD8^+^ effector memory T cells, as well as stronger *in vitro* CD8^+^ T cell responses to IL-6 activation. Whether and which of these associations are causally responsible for the response boost observed in CMV-positive young adults remain unknown, but the response boost observation is intriguing and may be a manifestation of heterologous T cell immunity or the presence of CMV-specific CD8^+^ T cells that crossreact against influenza [46], although there is scant evidence for such crossreactive T cells in CMV-positive subjects [41]. An equally compelling possibility, as suggested by the higher level of circulating IFN-γ in CMV-infected subjects [41], is that chronic CMV infection creates a ‘poised’ immune-response environment, akin to that induced by some vaccine adjuvants, to help enhance QR. How such enhancements wane in the elderly is again not clear. The general immunosenescent state, for example hyporesponsiveness of T and other immune cells, may alter the effects seen in older individuals. Chronic, persistent exposure to CMV over a longer period in the elderly may also lead to a state of immune exhaustion and reduction in T cell diversity [47] such that the effects of CMV infection on vaccination, if any, are convoluted with general age effects, thus rendering it undetectable in older subjects. It is also interesting to note that the young and the old in this cohort had similar frequencies of CMV infection, indicating that differences in infection status heterogeneity between the young and old cannot explain why correlation with CMV status can only be found in the young (see Figure 1B for effects of inter-subject variability on detecting correlations). Thus, chronic infection status can exhibit substantial heterogeneity in the population and is an important intrinsic correlate of vaccination outcome. Further global interrogation of these variables together with other immune parameters should lead to a better understanding of how they interact functionally with each other to influence immune responses in humans.

#### Gender and BMI

Other intrinsic variables such as gender – females generally tend to mount a stronger response than males – and BMI have also been shown to correlate with responses to vaccination [48–54]. Again, analyzing these variables together with a large number of immune parameters has proved to be informative. For example, by using regression analysis to identify statistically-interacting factors that correlate with seroconversion, the PBMC expression of a set of genes enriched for lipid biosynthesis was found to correlate with influenza vaccination response in a sex- and testosterone-dependent manner [51]: females with higher expression of these genes tend to generate a better response while the opposite is true in males, but only for those with higher levels of testosterone, otherwise the expression status of these genes seemed to have little effect on the male response. Interestingly, other differences between males and females, for example, those involving circulating inflammatory cytokines and the expression of genes on the Y chromosome, were not correlated with vaccine response. Whether lipid biosynthetic genes and related pathways play a direct, causal role in vaccine response is not clear because their status could simply reflect other immune states. Thus, broadly measuring immune parameters followed by modeling and analyses can reveal unexpected functional interactions between intrinsic biological variables and potential molecular markers of vaccine responsiveness.

#### Genetics

The genetic diversity of the human population provides a major source of variability and an important natural ‘perturbation’ useful in the study of human immunity. The effect of genetics on vaccination responses has been primarily assessed via twin and genome-wide association studies [31,55–59]. Earlier studies indicate that genetics play a substantial role in shaping vaccine responses: the estimated heritability (i.e., the amount of response variation that can be accounted for by genetics) ranged from 36% for hepatitis A vaccine to 89% for measles vaccine [31,56,60]. However, most recently a twin study that assessed a large number of immune parameters found little evidence of heritability in a substantial fraction of the assessed parameters, including serological response to influenza vaccination in adults [31]. While the relatively small size of the cohort may be a contributing factor, this divergence from previous observations could be age-related because most prior studies assessed vaccine-response heritability in infants and young children only. Consistent with this notion, Brodin *et al.* observed that some immune parameters, such as the frequency of circulating regulatory CD4^+^ T cells (Tregs), exhibit an age-dependent heritability trend: the correlation between monozygotic (i.e., genetically-identical) twins is higher in young as compared to older adults; the authors proposed that this could be because aging is correlated with increased, accumulated environmental exposures (e.g., infections) which can drive the immune system away from the largely genetically determined states seen during infancy. This hypothesis makes evolutionary sense because a genetically hardwired immune system would likely be less effective in constantly-changing environments.

The degree of measurable heritability and its relationship with variables such as age are likely parameter-, technology-, and cohort-dependent. For example, a large-scale genetic study involving more than 1600 subjects (14–102 years of age) found that the frequency of many circulating immune cell subsets [61], including Tregs and related subpopulations, exhibit substantial heritability. This was true even when using a more conservative, ‘narrow’sense definition of heritability in which only the contributions from additive genetic components were included (e.g., ignoring dominant and interacting genetic effects – see [62] for a discussion of these concepts and relevant references). Similarly, another recent twin study using female adults (40–77 years of age) found a broad tendency of circulating immune cell frequencies and cell surface marker expression to have detectable heritable components [58]. Thus, genetics can be a detectable, substantial contributor to the variation of immune parameters even in adults.

Quantitative comparison across studies and cohorts, however, can be challenging owing to differences in cohort characteristics (e.g., age, gender), environment exposure (e.g., children reared in the USA vs those reared in Sub-Saharan Africa), study design (twin vs family based), measurement technologies, parameters assessed, cohort size, as well as analytical approaches – for example, structural equation modeling (SEM [63]), Falconer’s formula (and SEM for a selected subset of traits), and variance decomposition using kinship information was employed in [31], [58], and [61] to estimate heritability, respectively. In general, analyzing cohorts with more genetic diversity (e.g., unrelated individuals) and less subject-to-subject variation in environmental exposure tend to result in a higher fraction of observed variation explainable by genetics. Whether twins are indeed comparable to the general population – a key assumption of twin studies [63] – is also not entirely clear. Twins are known to have lower birth weights and are more likely to be delivered via C-section, which can affect the initial microbiome compositions across body sites and thus immune functions [63–66]. Exact quantifications aside, these informative studies together support the notion that the immune state of a person at a given moment and subject-to-subject differences in a population emerge from the complex interplay between genetics and accumulated environment influences since birth (Figure 2A).

These findings also raise several fascinating questions. For example, how temporally stable are immune parameters in general? Those that are largely genetically-driven should be highly stable. Recently a study found that particular immune parameters including specific cell subset frequencies in blood exhibit seasonal fluctuations [67]. We found that many blood-based immune parameters are highly stable within a timescale of a few months during an active influenza season [13], but the timescales under which different immune parameters change temporally –and how those depend on the biological function and nature of the parameter – need to be further assessed in different types of subjects and environments. A more quantitative understanding of these issues can lead to a better selection of biomarkers (e.g., for predicting responsiveness before vaccination) by focusing on those that exhibit both high inter-subject variability and intra-individual temporal stability in the target population [13]. Furthermore, given that the immune system interacts with almost all organs and tissues, immune parameters can act as ‘modifiers’ to effect the ultimate phenotypic expression of many genetic variants. Such gene–immune environment interactions have yet to be systematically assessed, but they can account for a substantial portion of the missing heritability inherent across diverse health and disease phenotypes [68] and potentially help to explain the apparent paradox [69] that even highly-heritable immune-mediated diseases can be driven, at least partially, by non-heritable immune traits. Thus, achieving a more precise and predictive medicine as recently envisaged [34] would likely require the incorporation of extensive information on the state of the immune system.

Finding the actual genetic variants that underlie heritable immune traits can reveal genes and molecular networks that play important roles in shaping that trait, for example, in orchestrating responses to vaccination. Despite the substantial heritability of vaccine responses in the young (see above), only a few genetic variants, such as those in the human lymphocyte antigen HLA-DR locus [35] and a few other suggestive single-nucleotide polymorphisms (SNPs) [32,70], have been linked to vaccination responses. As discussed, this ‘missing heritability’ phenomena is commonly observed, likely because existing studies are underpowered for detecting associations involving variants with weak effects and genetic interactions among variants [71].

Perhaps as expected, linking variants to more ‘proximal’ traits (i.e., where the mechanistic path from genetic variation to the phenotype is relatively short) such as transcript levels in immune cells before and after activation [32,72,73], as well as cell surface marker expression and the frequency of circulating immune cell subsets, have yielded more associations [58,61]. Interestingly, some of these associations can be intricately linked. For example, a SNP in the locus nearby the *CD39* gene was strongly associated with CD39 expression in several cell subsets, including Tregs [58]. It turned out that this association could largely account for the observed association between this SNP and the frequency of CD39^+^ Tregs because average CD39 expression was correlated with the frequency of cells that fall inside the CD39^+^ gate. In general, a genetic variant can influence circulating cell subpopulation frequencies via several different potential mechanisms. As illustrated in the CD39 example, a variant can affect the expression of marker genes used to gate a cell subset. A variant can also regulate the proliferation and homeostasis of cell subsets to ultimately affect their frequencies in blood, for example, by changing the signal-sensitivity of a receptor that ‘listens’ to cues for cellular proliferation. In addition, a variant can regulate the trafficking of immune cells, such as by controlling the residence time of cells in tissues versus lymphoid organs versus the circulation. It remains to be assessed using bigger cohorts whether some of the genetic variants linked to these immune traits are also associated with vaccine QR. If so, employing techniques such as conditional independence tests can suggest whether the immune trait is a ‘mediator’ of the genetic effect, and is therefore potentially causal for determining vaccine QR [32,74].

## Pre-vaccination correlates and predictors

Despite their importance, intrinsic factors alone cannot solely account for the variability of many immune parameters. For example, many parameters measured before vaccination (i.e., a snapshot of the state of the immune system before the studied perturbation), (Figure 1A), had no apparent correlation to age, gender, and pre-existing antibody titers to influenza [13]; as discussed above, the genetic contribution to some immune parameters may also be low, especially in adults [31]. These observations are consistent with the notion that, in addition to intrinsic factors, the immediate environment, past exposure history, interactions among all of these factors, as well as temporal fluctuations driven by both deterministic and stochastic sources, together shape the observed state of the immune system at a given moment in time (Figures 1A,2). Thus, it is informative to ask whether there exist pre-vaccination correlates or predictors that can inform vaccination outcomes and thus also account for inter-subject variability beyond that attributable to observable intrinsic factors. Finding such predictive factors may also lead to clinically-useful molecular or cellular biomarkers for measuring immune heath as well as disease diagnosis and prognosis.

Using cross-validation and robust correlate analysis (Box 2) of PBMC gene expression, pathway ‘activity’ scores that substantially reduced the dimensionality of transcript-level information (Box 2), and the frequency of 120+ cell subsets in a healthy, largely young cohort (21–62 years of age; median age in the 20s), we found a set of pre-vaccination predictors and correlates that were independent of age, gender, and pre-existing serology [13] [in this case a titer response definition (called adjMFC) was used that was designed to remove the dependency of the titer response on initial serology (Box 2)]. Even in this cohort with mostly young adults, age effects on both pre- and post-vaccination parameters, including titer responses, were readily detectable. Interestingly, most of the predictive signal came from the frequency of a few cell subpopulations, including both memory B and T cell subsets, although robust correlates (that were not able to predict reliably – Box 2) involving gene expression and pathway activity were also uncovered. While PBMC gene expression can contain information about the frequency of its constituent cell subsets [75], here cross-validation based prediction could not be attained reliably using gene expression information alone, likely because statistical power was insufficient and the frequency of the predictive subsets was relatively low, and thus their variations were undetectable in the transcriptome of unfractionated PBMCs.

By utilizing data from multiple time-points (days −7, 0, and 70; day 70 was used because nearly all of the parameters that responded to the vaccine had reverted back to their baseline levels by then), we were able to decompose the total observed variation of a parameter into inter-versus intra-subject variations, with the latter covering a period of more than 2 months [13] (see Box 2 for a discussion of estimating inter- vs intra-subject variation in this manner). Many of the predictive cell populations were highly temporally stable and yet had substantial inter-subject variability, suggesting that they reflected immune states tightly controlled within a person, but yet had considerable diversity in the population we examined. Such parameters are ideal biomarker candidates not only because they likely capture personal biological states but also because their large inter-subject differences would render their measurement more robust against noise and timing of assessment. The heritability of and which genetic variants might regulate these parameters are not yet known. Although SNP data were also collected in this cohort, the cohort was too small to robustly power a genome-wide association analysis. A few of the predictive parameters exhibited less temporal stability (e.g., IL22^+^CD161^+^CD4^+^ T cells), but their removal only affected the prediction of an initial titer-dependent response metric (called MFC), suggesting that baseline titers might play a role in shaping these parameters.

One of the predictive cell subsets was effector memory CD4^+^ T cells (CD45RA^−^CD27^+^CCR7^−^) whose frequency negatively correlated with the adjMFC (initial titer-independent) metric (Box 2) [13]. This correlation could merely be a reflection of other mechanisms with indirect or no involvement of these cells, although a lower frequency of effector memory CD4^+^ T cells in the periphery could correspond to a higher level of central memory cells (i.e., those that are CCR7^+^), which might help B cells in secondary lymphoid organs to generate a more effective antibody response [76]. However, because antigen specificity was not assayed in these cells, it remains to be determined what fraction, if any, of these memory cells were influenza-specific. The potential mechanisms behind the other predictive cell subsets, including naïve, transitional, and memory B cell subsets that expressed CD38, are equally unclear. Again, it is unlikely that a sizable fraction of these cells were influenza-specific (given all the other antigens an individual would have encountered), even though influenza-specific memory T and B cells are known to be associated with titer responses [77,78]. Interestingly, the frequency of a related B cell subset (IgA^+^IgG^+^CD27^+^ switched memory B cells) was found in an earlier study as a pre-vaccination correlate of the fold-change in HI titers after influenza vaccination [79]. It would be interesting to determine whether the frequency of these switched memory B cells is related to the level of initial titers and to further assess the overlap among these emerging pre-vaccination B cell correlates.

Whether and which of the pre-vaccination correlates and predictors discussed above are cohort- or season-specific remains to be determined using independent, larger cohorts vaccinated in different seasons and geographic locations. An intriguing question is whether some of these parameters are not merely predictors of influenza vaccination responses but reflect the general propensity of the immune system to respond to perturbations. Answering these questions and finding additional pre-perturbation predictors of immune responses are crucial steps toward a more predictive, personalized, and precise assessment of health and disease [34].

## Response correlates and predictors

Despite extensive inter-subject heterogeneities at baseline in the values of intrinsic and other variables (e.g., those discussed above), coherent post-vaccination changes in PBMC or whole-blood gene expression, frequencies of cell subsets, and serum cytokines that are qualitatively consistent across a large fraction of the cohort can be detected in response to, for example, influenza, yellow fever, malaria, smallpox, and HIV vaccination [35]. The magnitude of these changes, however, tends to be highly variable across subjects and, again, these variations have empowered the discovery of vaccine-response correlates and predictors (Figure 1).

Post-vaccination predictors of antibody responses uncovered thus far include (i) the magnitude of increase in plasmablast frequencies and related genes (e.g., tumor necrosis factor receptor superfamily member 17, *TNFRSF17*), typically found on day 7, but sometimes also on day 3 post-vaccination (see below on a discussion on timing heterogeneity) [10,19,20,23], and (ii) the level of increase in interferon and inflammatory gene expression on day 1 [10,13,21,33]. As expected, given the key role plasmablasts play in antibody generation, the ability of the ‘plasmablast signature’ to predict the antibody titer response was consistently found across studies, including those involving the influenza, yellow fever, and conjugate meningococcal vaccines [33]. In addition to antibody responses, post-vaccination correlates have also been identified for CD8^+^ T cell responses, particularly for live, attenuated viral vaccines such as YF-17D [33,35]. Because several excellent reviews already cover post-vaccination correlates found in recent years using systems approaches (e.g., [30,33,35,80,81]), I will finish by briefly highlighting some emerging connections between intrinsic variables and the magnitude of post-vaccination changes in key parameters, such as those related to plasmablasts, as well as response timing heterogeneity.

Both age and gender can have an effect on the magnitude and timing of the plasmablast response. A recent study using PBMC transcript expression profiling found that the plasmablast response was either induced early on day 2 (instead of day 7) or not found at all in older adults on days 2, 4, 7, or 28 post-vaccination [82]. As expected, those without a plasmablast response did not have an increase in antibody titers. On the gender front, for example, YF-17D was shown to induce a significantly stronger early response involving antiviral, Toll-like receptor, and interferon-related genes in females than in males [48], providing suggestive mechanisms for why females tend to respond better to vaccines than males (see above). Similarly on the genetics side, Franco *et al.* identified SNPs that were significantly associated with and thus can influence gene expression changes on days 1 or 3 post influenza vaccination [32]; some of these SNPs and transcripts were also suggestively correlated with the antibody titer response. Thus, age, gender, and genetics can all shape the magnitude of early vaccination responses marked by gene expression. However, much remains to be discovered on these issues, particularly by assessing more parameters in larger cohorts with extensive variations in intrinsic factors.

In general, snapshot measurements of post-vaccination changes are subject to variations in response timing and duration. A recent study used daily gene expression measurements to show that the temporal pattern of B cell response signatures can be highly subject-specific [40]. For example, one subject displayed a peak plasma-blast response ~4 days post-vaccination, but a similar response did not emerge until the typical day 7 in another subject, albeit with greater duration; both subjects were thought to be mounting recall responses because both had pre-existing antibodies against influenza and had been vaccinated in previous seasons. Assessing a larger cohort of subjects is needed to determine whether such timing variation correlates with titer responses. Difference in the duration of the plasmablast response has also been observed between the pneumococcal and influenza vaccines [23]: although both responses peaked around day 7, the former mounted a substantially longer response lasting past day 10. Thus, the source of variability driving a predictive or correlative signal can be subtle: correlation involving the magnitude of a response measured at a particular time-point may reflect underlying variations in response timing or duration. Dense temporal measurements of diverse immune parameters in a large cohort of subjects before and after vaccination will be needed to better understand these issues.

## Concluding remarks

Several challenges need to be overcome by the research community to realize the full potential of the systems-based approaches discussed in this review (also see discussions in [2,33,35,83]). For example, while the throughput of data generation is increasing, with a continual drop in cost per sample, improvements in assay sensitivity as well as lowering of measurement noise and batch-to-batch variations are needed [35] to empower demanding statistical inference and, ultimately, mechanistic modeling [12] – in particular, to learn the network and quantitative rules of interactions among variables and how they work together to shape immune-response outcomes. Assay standardization and establishment of common ‘cores’ shared by laboratories and institutions are important to minimize technical variations and to ensure reproducibility and data reusability (e.g., [84,85]). Existing datasets also tend to have limited resolution, for example, measuring mixtures of cells (e.g., whole blood or PBMCs) across only a few time-points, whereas assessment of CD8^+^ T cell transcriptomes, as demonstrated by a recent study, enabled autoimmune disease-activity prognosis beyond that achievable via measuring the transcriptomes of whole blood or PBMCs [86]. Automating sample collection and processing using robotics [87] as well as miniaturizing assays (e.g., using microfluidics [88,89]) to reduce input amount requirements, for example, the minute amount of blood obtained from finger pricks [23], should lead to substantial increases in both throughput and resolution. Ultimately, as single cell ‘omics’ technologies – for example, for assessing the transcriptome [90,91], proteome [7,92], metabolome [93], and epigenome [94,95] – achieve greater sensitivity and affordability [8,96] we should be able to comprehensively interrogate the state of individual cells and eventually forgo the bulk measurement of cell populations. These developments should also help to overcome a major challenge on the computational front: the lack of statistical power in modeling the relationships among tens to hundreds of thousands of variables using only tens to hundreds of samples. Even when sample sizes increase dramatically, applications of meta-analysis and modeling approaches to integrate information from multiple studies and databases such as the NCBI Gene Expression Omnibus (GEO) (e.g., [10,97]) would continue to be an effective strategy to not only increase data resolution and statistical power but would also help to mitigate study and cohort-specific biases to obtain more robust biological insights.

Another major barrier, particularly for moving toward mechanistic modeling in humans, is the scarcity of information at the tissue level where much of the immune activities occur. While this will remain the case given the general lack of access to human tissues, advances in non-invasive imaging (e.g., [98,99]), assessment of postmortem tissues [100,101], further advances in animal models that mimic humans (e.g., humanized mice [102]), and a better quantitative understanding of correlations between immune parameters in blood and tissues, should help overcome these barriers.

Several types of variables that may be particularly relevant as predictors of vaccination outcome, but whose role in vaccination response orchestration is only beginning to be investigated at the systems level, include the composition of the gut microbiome and virome [103], as well as the variable-receptor repertoire of T and B lymphocytes. For example, the microbiota and their sensing via Toll-like receptor 5 (TLR5) were recently shown to be essential for productive antibody responses following influenza immunization in mice [104]. The post-vaccination B cell repertoire was more diverse – for example, more B cell clones were expanded – and exhibited higher levels of somatic hypermutation in high-than in low-responders [105]. Intriguingly, convergent antibody responses at the sequence level have been observed across subjects spanning different seasons [105], raising the prospect that such stereotypic responses to specific immune perturbations can be used to reconstruct the exposure history of an individual. Because past exposure is likely an important determinant of response quality to future perturbations such as vaccination (Figure 2A), such inferred histories can potentially be integrated computationally with other data modalities to enable better prediction of vaccination outcomes [106].

By continued utilization of the inherent biological variability of the human population spanning health and disease, as well as taking advantage of the inevitable increase in the scale, sensitivity, and resolution of measurement technologies, iterative applications of immune-state measurement and integrative computational modeling should yield an increasingly comprehensive catalog of vaccine- and immune-response correlates and predictors, as well as of the networks of functional interactions among them. As discussed, not only can such a catalog be useful to inform vaccine trials [30] and empower personalized strategies for disease prevention and treatment [34], it can be used to generate mechanistic hypotheses that may ultimately lead to better vaccine designs and a more quantitative understanding of how immune responses are orchestrated across space and time in humans – from molecules to cells to organs to the entire organism.
